# Endovascular treatment of ischemic large-vessel stroke due to infective endocarditis: case series and review of the literature

**DOI:** 10.1007/s10072-020-04599-9

**Published:** 2020-07-22

**Authors:** Lucio D’Anna

**Affiliations:** 1 Department of Stroke and Neurosciences, Charing Cross Hospital, Imperial College London NHS Healthcare Trust, Fulham Palace Road, London, W6 8RF UK; 2 Division of Brain Sciences, Imperial College London, London, UK

**Keywords:** Infective endocarditis, Stroke, Mechanical thrombectomy

## Abstract

**Background:**

Mechanical thrombectomy is the standard of care, in selected patients, for acute ischemic stroke with large vessel occlusion but its use in patients with stroke secondary to infective endocarditis is controversial. We report three cases of acute ischemic stroke treated by mechanical thrombectomy and we propose an extensive review of the literature to evaluate the clinical safety and efficacy of thrombectomy in patients with stroke secondary to infective endocarditis.

**Methods:**

A comprehensive literature search was performed following a pre-specified protocol of the Preferred Reporting Items for Systematic Reviews and Meta-Analyses statement. Case reports, cases series, cross-sectional studies, case control studies, randomized controlled trials or nonrandomized controlled trials were considered that included endocarditis-related acute ischemic stroke patients who underwent mechanical thrombectomy.

**Results:**

The database search yielded 431 relevant records published until January 2020. Nineteen articles fulfilled the eligibility criteria that described thirty patients. After the thrombectomy, 13.3% of the patients experienced intracranial haemorrhage. After the procedure, the median National Institutes of Health Stroke Scale score dropped from 15 (IQR 7) to 2.5 (IQR 5.75). At 90 days, mortality was 23.3% while 46.7% of the patients were functionally independent (mRS ≤ 2).

**Discussion:**

Based on our review, the use of mechanical thrombectomy in patients with large vessel occlusion due to endocarditis-associated stroke might improve patient outcome but it should be considered on a case by case base as the safety has not been well established yet. Further research on risk stratification is needed to drive clinician during the decision-making process.

## Introduction

Acute ischemic stroke is the most common neurological complication of infective endocarditis, manifesting clinically in 20–40% of the patients [1, 2]. Conversely, complications may be completely silent as asymptomatic ischemia can occur in another 30–40% of patients [3]. Patients with stroke secondary to infective endocarditis have a severe prognosis leaving only less than one-third of patients alive with functional independence [4].

Treatment of patients with acute stroke secondary to infective endocarditis is suboptimal as thrombolytic therapy is contraindicated due to high risk of haemorrhagic transformation of the infarct [5]. Mechanical thrombectomy is the standard of care, in selected patients, for acute ischemic stroke with large vessel occlusion. However, its efficacy and safety in patients with stroke secondary to infective endocarditis have limited evidence in the literature. Then, there is an urgent need of improved treatment for patients with stroke secondary to infective endocarditis.

We report here a case series of patients with stroke secondary to infective endocarditis treated with mechanical thrombectomy and a review of the current available literature on this issue.

## Methods

### Search strategy and study selection

**Fig. 1:**
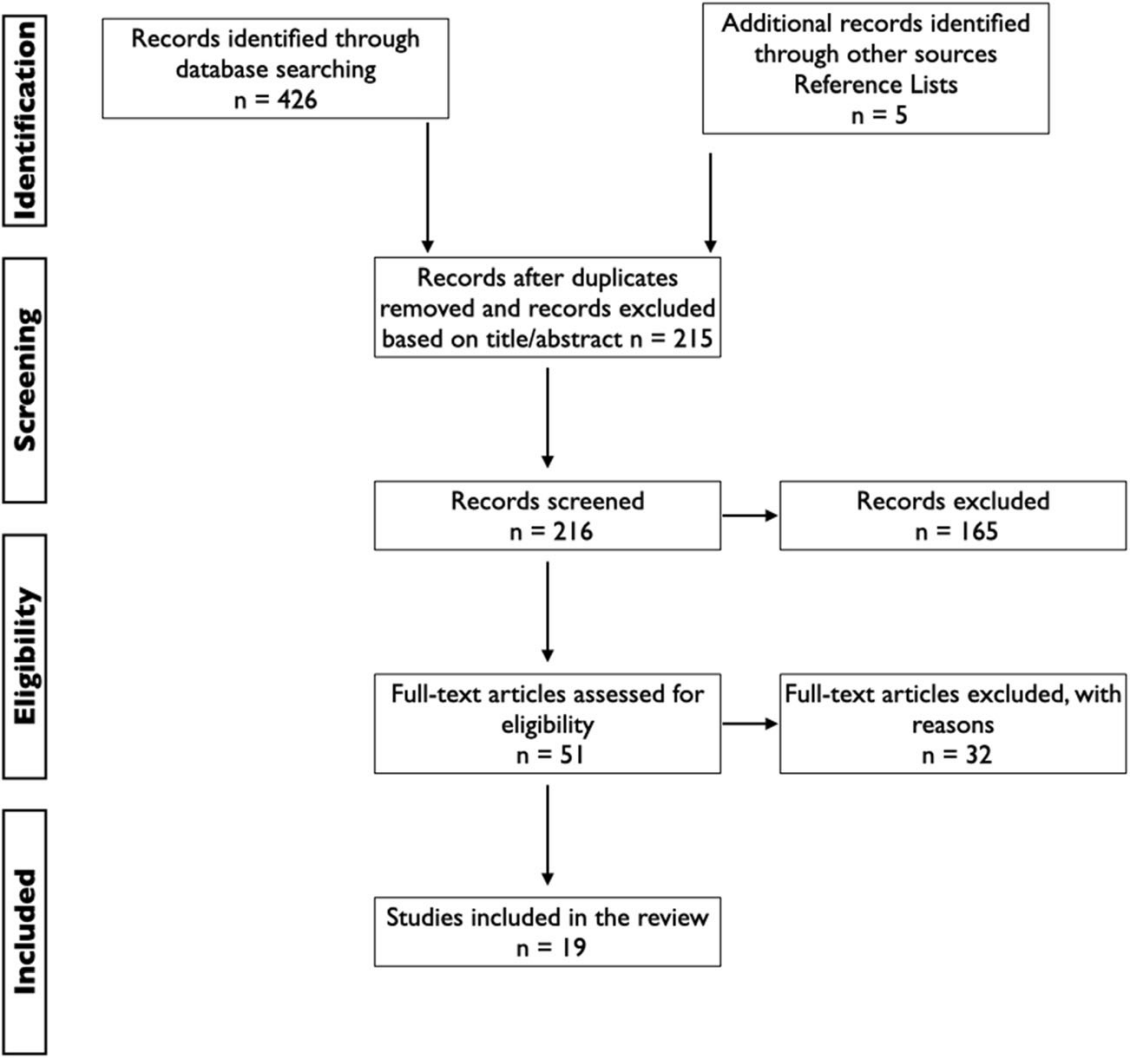
PRISMA flowchart

We performed a systematic review following PRISMA (Fig. 1) guidance [6]. Systematic search for the reports published until January 2020 was conducted in PubMed, Cochrane, SciELO, B-on, Google scholar and clinical trial registries for relevant articles. Reference lists from all included articles and abstracts were also assessed for any additional relevant studies not identified through the initial search. For those meeting the eligibility criteria, full-text articles were obtained. Pre-defined eligibility criteria were applied. For the search strategy, we combined the terms ‘stroke and endocarditis’ with ‘thrombectomy’. We did also the same search, but without the word ‘stroke’.

### Eligibility criteria

Only reports published in English were considered. We included randomized or nonrandomized controlled trials, case control studies, cross-sectional studies, case series and case reports that reported the treatment of patients (> 18 years old) with acute stroke secondary to infective endocarditis with mechanical thrombectomy. Title and abstracts of all retrieved articles were assessed for inclusion.

### Data collection process

We extracted the following information from the reports: age, gender, site of the infective endocarditis, presence or not of atrial fibrillation, treatment with oral anticoagulant or not, blood culture pathogen, baseline National Institutes of Health Stroke Scale (NIHSS) scores, treatment with intravenous thrombolysis and site of the large vessel occlusion. We also extracted as neuroradiological outcome the presence of intracranial haemorrhage after treatment and the thrombolysis in cerebral infarction (TICI) [7] scale score defined as grade 0, no perfusion; grade 1, penetration with minimal perfusion; grade 2A, only partial filling of the entire vascular territory visualised; grade 2B, complete filling of all the expected vascular territories visualised but the filling is slower than normal; and grade 3, complete perfusion. We documented the endovascular revascularization technique used, the NIHSS score after treatment and the modified Rankin Scale (mRS) at follow-up. Whenever any data is not provided, it was documented as not reported (NR).

### Risk of bias in individual studies

The quality of the articles was examined according to the Quality Assessment Tool for Case Series Studies of National Heart, Lung, and Blood Institute and to the Case Reporting Guidelines of Care (2013).

### Data synthesis

We summarised the data obtained in Table 1. In Table 2, we provided the % of patients with mRS score 0–1 and 0–2 at 90 days. We have also calculated the median (IQR) of the NIHSS at 24 h after the mechanical thrombectomy and the median (IQR) change in NIHSS score from baseline to 24 h after the procedure.

**Table 1:** Characteristics of patients with stroke and infective endocarditis treated with mechanical thrombectomy

Authors	Age/sex	Site of the IE	AF	Treatment of OAC	Blood culture pathogen	Onset NIHSS score	Treatment with IV thrombolysis	LVO site	TICI	Endovascular revascularization technique	ICH	NIHSS outcome	mRS at follow-up
D’Anna	67/M	Mitral valve	No	No	*Gemella morbillorum*	17	No	M1	3	Aspiration	No	0–24 h after	0 at 90 days
D’Anna	30/F	Aortic valve	No	No	*Neisseria gonorrhoeae*	22	No	Distal ICA	2a	Aspiration and stent retriever	Yes	22–24 h after	3 at 90 days
D’Anna	65/F	Mitral valve	No	No	*Staphylococcus aureus*	18	NR	M2	0	Aspiration	No	21–24 h after	4 at 90 days
Sloane et al.	59/F	Mitral valve	No	No	Negative	21	No	M1	2B	Stent retriever	No	6 after the procedure	1 at 180 days
Distefano et al.	75/M	Aortic valve	No	No	*Enterococcus faecalis*	16	Yes	M1	NR (recanalised)	Aspiration	Yes	NR	6 few days later
Sgreccia et al.	31/M	Aortic valve	No	No	*Candida parapsilosis*	18	Yes	ICA bifurcation	3	Stent retriever	No	0–48 h later	0 at 90 days
Ambrosioni et al.	79/M	Prosthetic (mechanical)	NR	Yes	*Staphylococcus aureus*	9	NR	M1 and ICA	0	Stent retriever	No	35–24 h after	6 at 7 days
Ambrosioni et al.	69/ F	Prosthetic (mechanical)	NR	Yes	*Streptococcus oralis*	10	NR	Basilar	3	Stent retriever	No	2–24 h after	0 at 7 and 90 days
Ambrosioni et al.	56/F	Native	NR	No	Negative culture	19	NR	M1	3	Stent retriever	No	2–24 h after	0 at 7 and 90 days
Ambrosioni et al.	72/M	Native	NR	No	*Streptococcus dysgalactiae*	35	NR	Basilar	3	Stent retriever	No	35–24 h after	6 at 7 days
Ambrosioni et al.	79/F	Prosthetic (biological)	NR	Yes	Negative culture	5	NR	M1	2B	Stent retriever	No	2–24 h after	2 at 7 and 90 days
Ambrosioni et al.	85/M	Prosthetic (biological)	NR	No	*Staphylococcus epidermidis*	8	NR	M1	3	Stent retriever	No	0–24 h after	0 at 7 days and 6 at 90 days
Bolognese et al.	42/M	Aortic valve	No	No	*Streptococcus viridans*	3	No	M2	2B	Aspiration	No	0 at 4 weeks	NR
Elodie et al.	70/ F	Aortic valve	Yes	Yes	Negative culture	10	No	M1	NR (recanalised)	Stent retriever	No	1 immediately after	1 at 90 days
Nishino et al.	72/M	Mitral valve	Yes	Yes	*Streptococcus salivarius*	NR	No	M2	NR (recanalised)	Stent retriever	No	NR	6 at 9 days
Scharf et al.	NR/NR	Mechanical mitral valve and native aortic valve	No	Yes	*Streptococcus*	12	No	M1	3	Stent retriever and aspiration	No	1	0 at discharge
Sveinsson et al.	33/M	Prosthetic mitral valve	No	Yes	*Serratia marcescens*	14	No	M1	NR (recanalised)	NR	No	1 at discharge	1 at discharge
Sveinsson et al.	67/M	Prosthetic mitral valve	Yes	Yes	*Enterococcus faecalis*	13	No	M1	NR (recanalised)	NR	No	3 at discharge	1 after few months
Sveinsson et al.	39/F	Mitral valve	No	No	NR	15	No	M2	NR (recanalised)	NR	No	4 at discharge	2 after 3 months
Ladner et al.	40/NR	Aortic valve	No	No	*Enterococcus faecalis*	3	No	M1	3	Aspiration	No	0 at 13 days	0 at 13 days
Kim et al.	40/F	Mitral valve	No	No	*Streptococcus mitis*	15	No	M2	NR (recanalised)	Aspiration	No	3 at 2 days	2 at 3 months
Toeg et al.	73/M	Bioprosthetic aortic valve (replaced 8 weeks prior)	No	No	Gram-positive cocci	20	No	M1, A1 and distal ICA	NR (recanalised)	NR	No	2 immediately after; 0 after 8 months	NR
Akkoyunlu et al.	23/F	Mitral valve	No	No	Gram-positive coccobacillus	NR	No	M1	NR (recanalised)	NR	No	NR	NR
Kang et al.	39/F	Mitral valve	No	No	*Streptococcus gordonii*	16	No	M1	2b	Stent retriever	No	3 at 4 weeks	NR
Dababneh et al.	67/F	Bovine mitral valve (replaced 6 months prior)	Yes	Yes	Gram-negative vancomycin-resistant rods	NR	No	Between segments M1 and M2	2 or 3	Stent retriever	No	NR	6 at 7 days
Kan et al.	78/F	Aortic valve	No	No	No	16	No	M2	3	Stent retriever	No	12–24 h later	NR
Liang et al.	70/F	Mitral valve	Yes	Yes	Group B *Streptococcus agalactiae*	24	No	M2	NR (recanalised)	Stent retriever	No	NR, reported no residual neurology	NR
Walker et al.	NR/NR	NR	NR	NR	Coagulase-negative Staphylococcus	14	Yes	NR	NR	Stent retriever	Yes	NR	6
Walker et al.	NR/NR	NR	NR	NR	*Enterococcus faecalis*	14	Yes	NR	NR	Stent retriever	Yes	NR	6
Bain et al.	24/F	NR	No	Yes	Gram-positive bacilli	18	No	M1	NR (recanalised)	Stent retriever	No	7–24 h after; 2 after 2 months	NR

*M* male; *F* female; *IE* infective endocarditis; *IV* intravenous; *AF* atrial fibrillation; *OAC* oral anticoagulant; *NIHSS* National Institutes of Health Stroke Scale; *ICA* internal carotid artery; *LVO* large vessel occlusion; *TICI* thrombolysis in cerebral infarction; *ICH* intracranial haemorrhage; *mRS* modified Rankin Scale; *NR* not reported

**Table 2:** Efficacy outcome

mRS score at 90 days	IE patients treated with MT
mRS score 0–1 at 90 days	36.7% (11/30)*
mRS score 0–2 at 90 days	46.7% (14/30)*
NIHSS at 24 h	
Median score	2.5 (IQR 5.75)
Change in NIHSS score from baseline to 24 h	
Median change	− 14 (IQR 10)

*MT* mechanical thrombectomy; *IE* infective endocarditis; *NIHSS* National Institutes of Health Stroke Scale; *mRS* modified Rankin Scale; *IQR* interquartile range

*mRS score at 90 days is not available for seven patients

## Case series

### Case 1

A 30-year-old female patient was brought to our emergency department 4 h after she developed right-sided hemiparesis, aphasia, right homonymous hemianopia, right central facial paralysis and reduced sensation in the right side of the body while she was at the gym (NIHSS 22). A month earlier, she presented to her local general practitioner (GP) with symptoms including sore throat, lethargy and general malaise. Her GP found no specific abnormalities on thorough general examination. CT of the brain showed already established changes in the left middle cerebral artery territory with an Alberta stroke program early CT score (ASPECTS) of 6. CT angiography of the brain showed a thrombus in the terminal segment of the left internal carotid artery (ICA). Thrombolysis was not performed because of the low ASPECT score on the CT brain. Mechanical thrombectomy was performed using a combination of aspiration and stent retriever, and partial recanalization was obtained (TICI 2a). A day later, the CT of the brain showed haemorrhagic transformation with an intraparenchymal haematoma centred in the left lentiform nucleus and her NIHSS was unchanged. At the same time, she developed rising fever and elevated CRP (145 mg/L). On day 4 of her admission, transthoracic echocardiogram showed vegetation on the aortic valve with associated severe aortic regurgitation. Left ventricular function was unimpaired. Blood cultures were positive for Gram-negative diplococcus, identified as *Neisseria gonorrhoeae*. She was therefore treated with ceftriaxone and azithromycin. After 3 months, her mRS was 3.

### Case 2

**Fig. 2:**
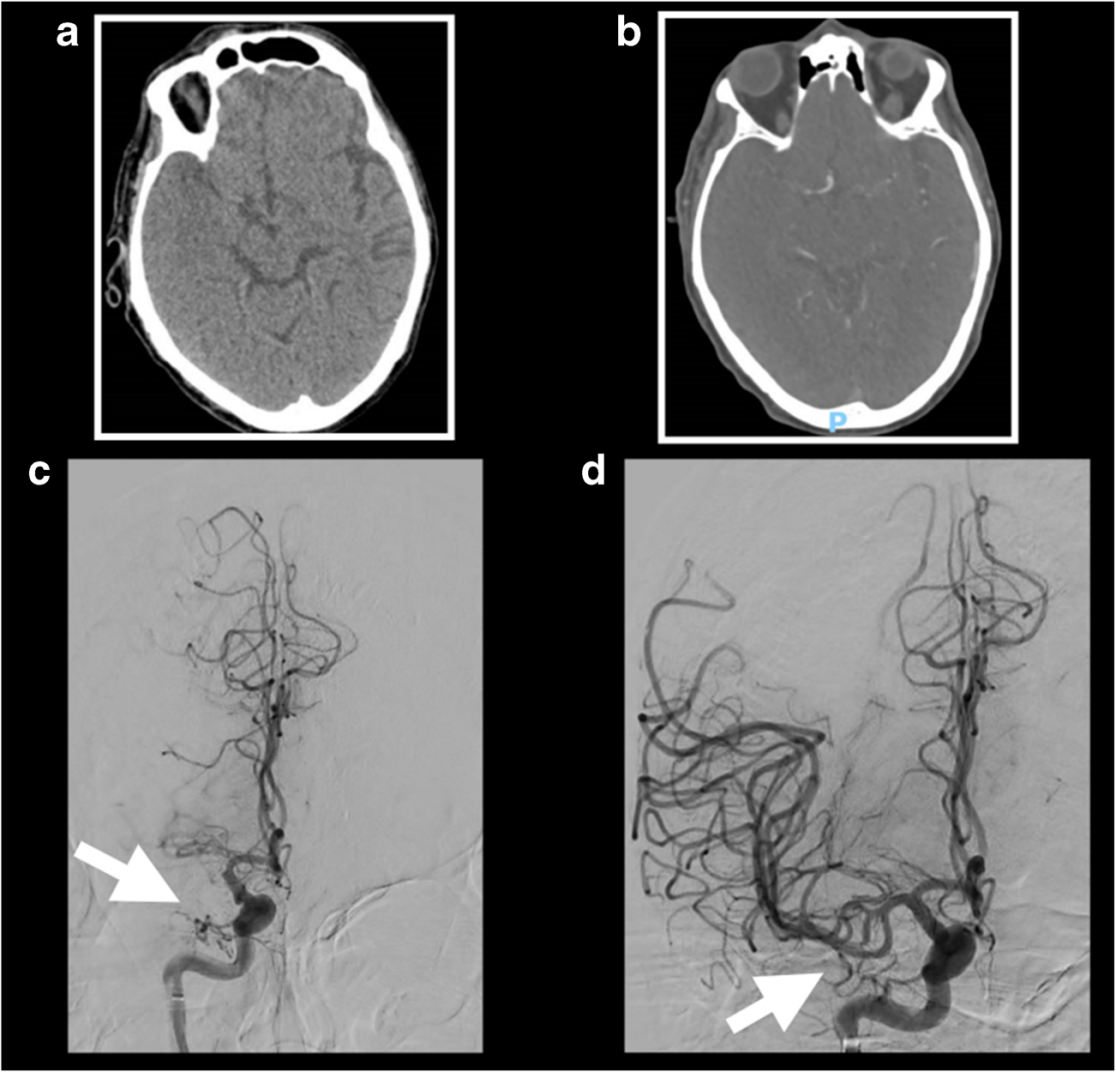
Case 2. **a** Axial CT head showing hyperdensity of the M1 segment of the right middle cerebral artery consistent with acute thrombus, and focal hypodensity of the right temporal lobe with loss of grey-white matter differentiation. **b** CT angiography demonstrating abrupt cutoff at the proximal M1 segment of the right middle cerebral artery. **c** Angiography confirmed occlusion of the right middle cerebral artery. **d** Post-angiographic images demonstrating the restoration of the flow in the right middle cerebral artery following successful thrombectomy

A 67-year-old male patient was transferred from a local hospital to our emergency department 3 h after sudden onset of left sided weakness, sensory disturbance and dysarthria. His NIHSS was 17. The patient was initially admitted for infective endocarditis of his native mitral valve and he was supposed to valve replacement surgery soon. Blood culture was positive for *Gemella morbillorum*. Computer tomography (CT) with CT angiography showed an occlusion of the M1 segment of the right middle cerebral artery. ASPECT score was 8 (Fig. 2a and b). Because of the evidence of endocarditis, intravenous thrombolysis was not considered an option. However, based on the large vessel occlusion, mechanical thrombectomy was performed under local anaesthetic with sedation. Endovascular thrombectomy was then performed with successful aspiration of the clot (TICI 3) (Fig. 2c and d). No immediate complications were recorded after the procedure. After 24 h, his NIHSS dropped to 13. Mitral valve replacement surgery was successfully performed 1 month later. At neurological follow-up after 3 months, the patient showed no neurological deficits (NIHSS 0) and mRS score of 0.

### Case 3

A 65-year-old female patient with staphylococcal native mitral valve infective endocarditis had a witnessed onset of right-sided hemiparesis and aphasia (NIHSS 18). The patient was initially admitted in a local hospital for infective endocarditis and treated with teicoplanin and gentamicin. The patient was brought to our emergency department 3 h and 45 min after she developed her stroke symptoms. Her past medical history included also liver cirrhosis, type 2 diabetes, hypertension and breast cancer. CT angiography showed an occlusion of both M2 divisional branches. Intravenous thrombolysis was contraindicated because of the evidence of endocarditis. Mechanical thrombectomy was considered due to the large vessel occlusion. Despite several attempts at aspiration, the clot remained in situ with the final angiography demonstrating a subtotal occlusion of both the M2 divisional branches (TICI 1). After 24 h, her NIHSS was 21 and the CT showed no haemorrhagic transformation. At neurological follow-up after 3 months, her mRS score was 4.

## Results

The database search yielded 431 relevant records published until January 2020. Nineteen articles fulfilled the eligibility criteria [8–26]. Three articles (16%) were case series while sixteen (84%) were single case report.

### Synthesis of results

Table 1 shows the characteristics of patients with stroke and infective endocarditis treated with mechanical thrombectomy. The median age of the patients was 67 years old (IQR 32.75). For three patients, their age was not reported. Eleven patients were men (36.7%) while for four patients (13.3%), the gender was not documented. The median baseline NIHSS resulted to be 15 (IQR 7). The most common site of the large vessel occlusion was the M1 (56.7%). After the thrombectomy, nine patients (30%) had TICI score of 3 while the TICI score of 2b was obtained in four patients (13.3%). After the treatment, four patients (13.3%) experienced intracranial haemorrhage. After the procedure, the median NIHSS dropped to 2.5 (IQR 5.75) (Table 2).

**Fig. 3:**
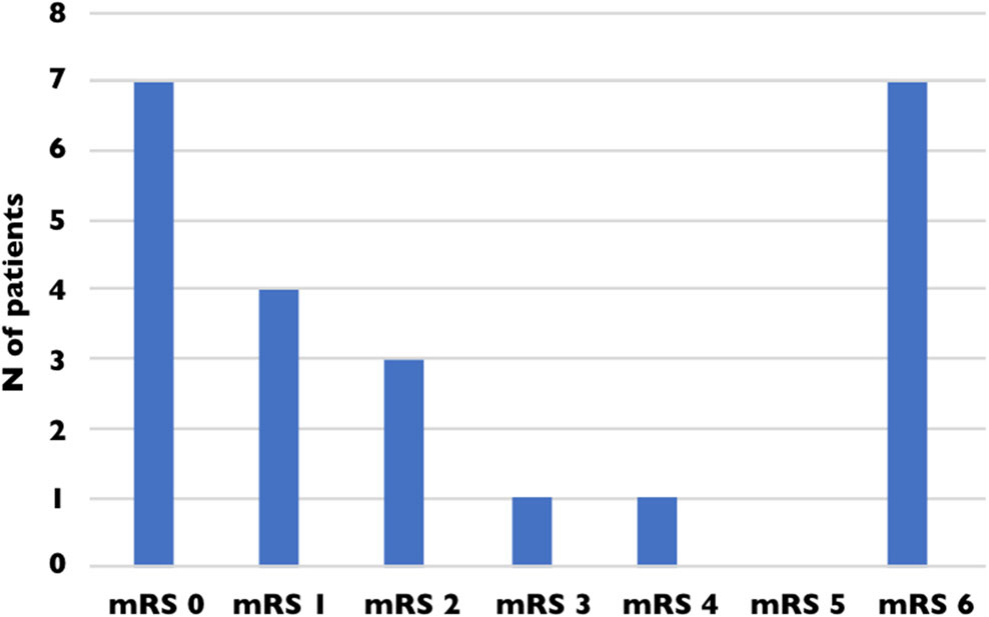
Distribution of the mRS at 90 days. mRS score at 90 days is not available for seven patients

After a follow-up of 90 days, seven patients were dead (mRS = 6) (23.3%) while fourteen (46.7%) were functionally independent (mRS ≤ 2) (Table 2) (Fig. 3). Of note, mRS score at 90 days was not available for seven patients.

## Discussion

Our review described the clinical presentation, management and outcome of patients treated with mechanical thrombectomy for thrombotic stroke due to infective endocarditis. Although mechanical thrombectomy is not recognised as the standard of care for acute stroke secondary to infective endocarditis, this review provides evidence of consistent benefit for endovascular treatment on disability in these patients. Indeed, in our review, 36.7% of the patients with stroke due to infective endocarditis treated with mechanical thrombectomy had a 90-day mRS ≤ 1 while 46.7% of the patients had a mRS ≤ 2 at 90 days. Overall, almost half of the patients with stroke due to infective endocarditis treated with mechanical thrombectomy were functionally independent after 3 months. Many previous studies showed that the occurrence of neurologic complications during infective endocarditis were associated with an increased risk of mortality [1, 2, 4, 27, 28]. More specifically, Thuny et al. [29] demonstrated that the risk of death differed according to the type of cerebrovascular disease. Patients with silent brain infarcts or transient ischemic attack had a better prognosis, whereas large stroke resulted to be a strong predictor of mortality independently of the other prognostic factors. Moreover, the cause of death in these patients was a direct consequence of this neurologic event. One explanation of this finding is the fact that the absence of large brain injuries carried a better prognosis and allowed early surgery to be performed with a low operative risk. This result underlines the critical need of safe reperfusion therapy for acute ischemic stroke due to infective endocarditis for salvaging ischemic brain that is not already infarcted to avoid dramatic cerebral damages.

To date, treatment with intravenous alteplase is not recommended for patients with acute ischemic stroke and symptoms consistent with infective endocarditis because of the increased risk of intracranial haemorrhage [30]. Despite the fact that the fibrinolysis might promote reperfusion through cerebral vessels occluded by septic emboli, histopathological studies suggested that cerebral infarcts caused by septic emboli are particularly prone to haemorrhagic transformation as a result of septic arteritis with erosion of the arterial wall in the recipient vessel, with or without the formation of mycotic aneurysms. The use of mechanical thrombectomy for ischemic stroke with large vessel occlusion due to septic emboli has been documented in few patients in literature so far [28]. In our review, we found that only 13.3% of the patients with endocarditis-related acute stroke treated with thrombectomy suffered an intracranial haemorrhage after the treatment. Bettencourt et al. [28] showed that the use of intravenous alteplase alone or combined with mechanical thrombectomy was associated with a 4-fold increased risk of intracranial haemorrhage compared with mechanical thrombectomy alone. The severe bleeding complications associated with the use of intravenous alteplase may suggest that mechanical thrombectomy alone should be considered in selected patients with infective endocarditis if there is a documented large vessel occlusion.

The patterns of endocarditis-associated stroke observed in previous studies are very heterogenous. Previous studies, using conventional and DWI MRI [31] [32, 33], showed that patients with acute ischemic stroke and infective endocarditis can have a variety of ischemic lesions, including single cortical, territorial, disseminated punctate and disseminated small and large lesions in multiple vascular territories. Infarction of the middle cerebral artery territory resulted to be the most common anatomical lesion, with involvement of the middle cerebral artery tree [31]. In this review, 93.3% of the patients (28/30) treated with mechanical thrombectomy had a large vessel occlusion in the anterior circulation. Previous studies proved the value of thrombectomy in anterior circulation acute ischemic stroke within the first 6 h of symptom onset [34–36]. In MR CLEAN [34], 33% of patients achieved a good clinical outcome being functionally independent with thrombectomy versus 19% with medical therapy; in EXTEND-IA [35], the respective outcomes were 71% versus 40%; in SWIFT PRIME [36], they were 60% versus 35%. Recently, two recent multicentre randomized controlled trials of mechanical thrombectomy of the anterior circulation initiated at a later time windows of up to 16 h and 24 h from symptom onset have shown that endovascular therapy is safe and highly effective in carefully selected patients with advanced imaging in comparison with medical management alone [37, 38]. The results of our review suggest that the use of thrombectomy might be an efficient and safe treatment for patients with acute large vessel occlusion of the anterior circulation associated with endocarditis and might help improve outcome; however, age, time from symptom onset, clinical severity of stroke symptoms, pre-stroke level of functioning and anatomic location of the large vessel occlusion are the most important determinants of candidacy for mechanical thrombectomy in this cohort of patients.

This study has different limitations. In first instance, our search did not find any randomized controlled trials to investigate the efficacy and safety of the mechanical thrombectomy in patients with acute stroke due to infective endocarditis as our review was based only on single case reports or case series. Secondly, there were some data missing and this made difficult to compare the results. Finally, we draw our conclusions in favour of the use of thrombectomy based on a potential publication bias.

In conclusion, based on our review, the use of mechanical thrombectomy in patients with large vessel occlusion due to endocarditis-associated stroke should be considered on a case by case base as the safety has not well established yet. Further research on risk stratification is needed to drive clinician during the decision-making process.

## Data Availability

The data that support the findings of this study are available from the corresponding author, LD, upon reasonable request.
